# Evolving Burn Care: The Transition from Life Preservation to Life Restoration―A Narrative Review

**DOI:** 10.3390/jcm15083102

**Published:** 2026-04-18

**Authors:** Tobias Niederegger, Jule Brandt, Thomas Schaschinger, Alen Palackic, Valentin Haug, Felix Klimitz, Ulrich Kneser, Christoph Hirche, Benjamin Ziegler, Martin Aman, Leila Harhaus-Wähner, Gabriel Hundeshagen

**Affiliations:** 1 Medical Faculty, University of Heidelberg, 69120 Heidelberg, Germany; tobias.niederegger@stud.uni-heidelberg.de (T.N.);; 2 Department of Hand-, Plastic and Reconstructive Surgery, Microsurgery, Burn Trauma Center, BG Trauma Center Ludwigshafen, University of Heidelberg, 67071 Ludwigshafen, Germany; 3 Department of Plastic and Reconstructive Surgery, Vivantes Hospital Friedrichshain, 10249 Berlin, Germany; 4 Department of Plastic Surgery, Hand and Reconstructive Microsurgery, Hand Trauma and Replantation Center (FESSH), BG Unfallklinik Frankfurt, Affiliated Hospital of Goethe University Frankfurt, 60389 Frankfurt, Germany; 5 Department of Hand-, Replantation- and Microsurgery, Department of Burns and Plastic Surgery, BG Klinikum Unfallkrankenhaus Berlin, Chair of Hand-, Replantation- and Microsurgery, Charité Universitätsmedizin Berlin, 12683 Berlin, Germany

**Keywords:** burn, burn injury, trauma, orthopedic trauma, intensive care

## Abstract

Over the past years, burn care has evolved from a discipline focused on survival to one centered on restoring long-term health, function, and quality of life. Significant advances in critical care, early excision and grafting, infection control, and metabolic support have transformed survival outcomes for even the most severe injuries. As a result, the field now faces a new frontier: understanding and managing the long-term physical, psychological, and systemic sequelae of survival. This review traces the evolution of burn care over the last century and outlines the challenges and priorities for the next 25 years. The first era of progress, defined by innovations in resuscitation, surgery, and critical care, has given rise to a growing cohort of long-term survivors. Research over the past decade has revealed that major burns induce chronic multisystem alterations, including metabolic, cardiovascular, neurocognitive, and immunological dysfunctions. Emerging concepts such as burn-associated heart failure exemplify this shift from acute to chronic disease understanding. Looking ahead, the future of burn medicine lies in personalized and lifelong care, supported by translational research, digital health, regenerative therapies, and interdisciplinary collaboration. Overall, burn care stands at a pivotal crossroads. By integrating precision medicine, rehabilitation science, and psychosocial care, we aim to move the field from survival toward sustained, holistic recovery over the next 25 years.

## 1. Introduction

The history of burn care is an impressive showcase of the dynamic evolution in modern medicine, transforming from a discipline defined by the fight for survival to one that seeks to restore health, function, and quality of life across the patient’s entire lifespan.

A lack of understanding of the underlying pathophysiology, inadequate infection control, and limited therapeutic options characterized early burn management [1,2,3]. Mortality was the benchmark criterion for extensive burns, and the focus of care was primarily on palliation and prevention of immediate complications [1]. The introduction of fluid resuscitation protocols in the mid-20th century, antibiotic therapy, and advances in anesthesia and surgical techniques, particularly early excision and grafting, marked a fundamental turning point. These milestones founded modern acute burn care, allowing patients with previously unsurvivable injuries to overcome the acute phase of severe burns [4].

As survival rates improved, a paradigm shift occurred in the philosophy and objectives of burn treatment. The historical success of acute burn therapy, through better resuscitation, critical care, and infection control, created a new reality: survival was no longer the endpoint, but the beginning of a complex, lifelong process. Clinicians and researchers increasingly recognized that many survivors faced severe physical and psychosocial sequelae that could persist for decades. These included chronic pain, hypertrophic scarring, contractures, thermoregulation disorders, and neuropathic complications, as well as profound psychological distress such as anxiety, depression, and post-traumatic stress disorder [5].

This awareness catalyzed a shift from a purely survival-oriented model to a holistic, patient-centered approach that integrates acute management with long-term rehabilitation, reconstructive strategies, and psychosocial support. In parallel, burn surgery has increasingly come to encompass the full armamentarium of plastic and reconstructive surgery, guided by reconstructive ladder principles—from staged scar release and contour reconstruction to local/regional flaps and microsurgical free tissue transfer for complex functional restoration and durable coverage [6]. The success of acute therapy exposed the urgent need to address the quality of survival, to rebuild not only the body but also the person behind the injury [7]. Consequently, interdisciplinary care teams now extend beyond surgeons and intensivists to include psychologists, physiotherapists, occupational therapists, social workers, and pain specialists. Rehabilitation and reintegration, once peripheral considerations, have become essential outcomes of modern burn care [8,9].

At the same time, advances in regenerative medicine, scar modulation, and digital health technologies are redefining what long-term recovery can look like [10]. Tele-rehabilitation, virtual reality-based pain therapy, and novel biomaterials are reshaping the continuum of care far beyond hospital discharge. This ongoing transformation highlights that the burn injury must now be understood as a chronic condition, requiring long-term management strategies tailored to the evolving needs of survivors [11,12].

Therefore, the present review aims to build a conceptual bridge between the past and the future of burn care, between the milestones that defined the past century and the challenges that will shape the next 25 years. By tracing the evolution from acute survival to comprehensive recovery, this article seeks to contextualize the paradigm shift that has redefined burn medicine: from saving lives to restoring lives.

## 2. The Past Century: Milestones in Acute Burn Medicine

### 2.1. Improved Survival Rates: A Multidisciplinary Achievement

Over the past century, acute burn care has undergone unprecedented progress, driven by a deeply interdisciplinary collaboration between intensive care medicine, surgery, anesthesiology, infectious diseases, and nursing. The remarkable improvement in survival rates is not the result of a single breakthrough but rather the cumulative effect of numerous, carefully refined treatment components [13,14]. Advances in general intensive care, particularly in hemodynamic monitoring, fluid resuscitation, and sepsis prevention, have fundamentally transformed the stabilization of critically burned patients. The introduction of goal-directed fluid therapy, early enteral nutrition, lung-protective ventilation, and continuous renal replacement techniques has refined physiological support, reducing secondary organ dysfunction and translating into markedly improved survival outcomes [15,16,17,18].

At the same time, the principle of early excision and grafting revolutionized surgical burn management. The early removal of necrotic tissue combined with immediate wound coverage significantly reduced infection rates, systemic inflammation, and the incidence of multiorgan failure. This surgical paradigm, coupled with advances in perioperative support, remains one of the greatest milestones of modern burn medicine [19].

Equally transformative has been the growing understanding of the pathophysiological mechanisms underlying the acute systemic burn disease. Insights into the hypermetabolic response, the complex release of inflammatory mediators, and the profound immunological dysregulation following thermal injury have laid the foundation for more targeted interventions [20,21]. Integrating these insights into clinical practice, through individualized nutritional therapy, modulation of the immune response, and optimized thermoregulation, has been key to reducing acute mortality and improving early outcomes.

### 2.2. The New Patient Cohort of Long-Term Survivors

As acute mortality has steadily declined over the past decades, the epidemiological landscape of burn medicine has undergone a fundamental transformation. What was once a field dominated by high mortality has now evolved into one characterized by high morbidity, a shift that reflects both the success of acute interventions and the emergence of new long-term challenges. The population of burn patients has changed accordingly: from those fighting for survival in the acute phase to a growing cohort of long-term survivors who now stand at the center of clinical, rehabilitative, and social focus.

This new reality has brought with it a complex burden of chronic sequelae. Many survivors live with persistent pain, hypertrophic scarring, contractures, neuropathic dysfunction, and psychosocial impairments such as depression, anxiety, and post-traumatic stress disorder. The physical recovery achieved in the intensive care unit often marks only the beginning of a prolonged rehabilitation journey that extends over years, sometimes decades [22].

**Figure 1:**
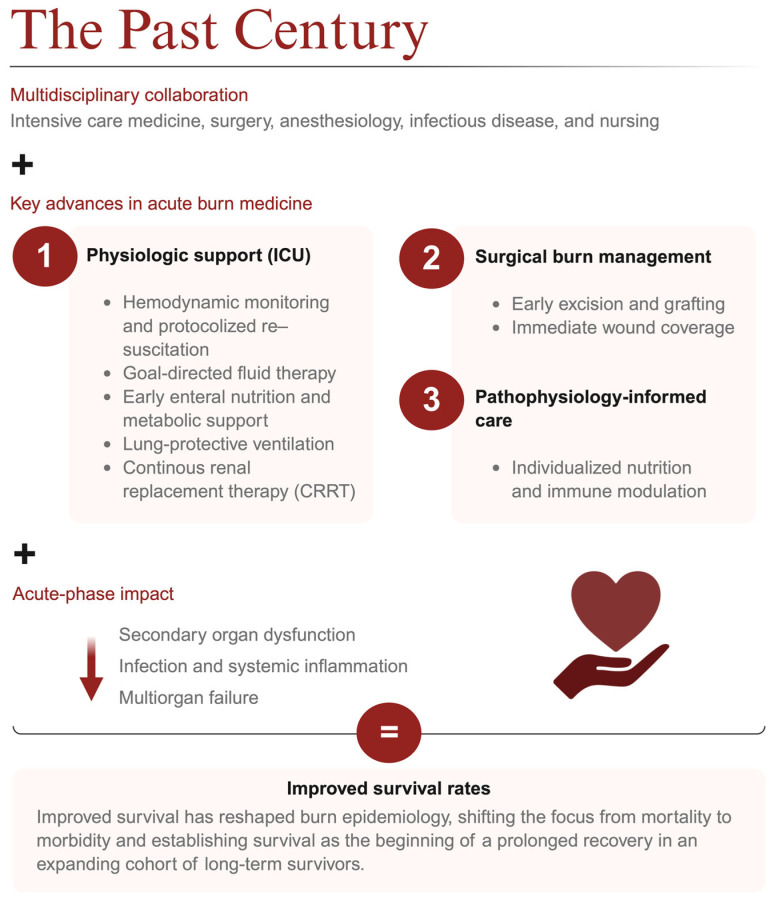
Over the last century, multidisciplinary burn care advances in ICU physiologic support, surgical management (early excision/grafting, wound coverage), and pathophysiology-informed care have reduced organ dysfunction, infection/inflammation, and multiorgan failure—leading to improved survival and greater emphasis on long-term recovery.

Epidemiological data from national and international burn registries consistently demonstrate an increase in the number and longevity of burn survivors, accompanied by a corresponding rise in the complexity and duration of post-acute care needs [23,24]. In Germany, the BG Trauma Centers (Berufsgenossenschaftliche Kliniken) play a leading role in meeting these demands. As one of the country’s foremost providers of specialized burn care, these centers serve as interdisciplinary hubs that aim to integrate acute stabilization, reconstructive and regenerative surgery, long-term rehabilitation, and psychological reintegration under one continuum of care [25,26]. Their coordinated, multidisciplinary approach is one example of how structural specialization and system-level organization have become key determinants of successful outcomes in the modern era of burn medicine (Figure 1) [27,28].

## 3. The Last 10 Years: Discovery of Long-Term Sequelae

### 3.1. Systemic and Organ-Specific Late Effects

In the past decade, burn medicine has entered a new phase of understanding, one that extends beyond survival and acute recovery to explore the systemic and long-term consequences of severe burn injury. As survival rates improved, it became increasingly apparent that major burns are not isolated traumatic events but rather chronic conditions with persistent, multisystemic alterations. Research has revealed that burn survivors often experience enduring endocrine–metabolic, cardiovascular, musculoskeletal, neuropsychological, and immunological changes that may last for years or even decades after the initial injury [20,21].

These alterations include sustained hypermetabolism, insulin resistance, and altered lipid metabolism; cardiac remodeling and reduced myocardial function; loss of muscle mass and bone density; as well as chronic inflammation, neurocognitive impairment, and psychiatric comorbidities [29,30,31]. Post-burn hypermetabolism is characterized by a prolonged increase in resting energy expenditure driven by persistent catecholamine release, inflammatory signaling, and endocrine dysregulation. Clinically, this response promotes ongoing protein catabolism, loss of lean body mass, impaired glucose handling, and delayed functional recovery, thereby contributing substantially to long-term morbidity even after apparent wound healing [32]. Together, these findings have redefined the understanding of burns as a systemic disease with long-term organ dysfunction, challenging the traditional perception of the burn injury as an acute, time-limited insult.

The growing recognition of these long-term sequelae has catalyzed the development of translational research approaches aimed at uncovering the underlying molecular and physiological mechanisms of long-term effects. Therefore, over the last decade, multidisciplinary collaborations have emerged between basic scientists, clinicians, and rehabilitation specialists to bridge laboratory discoveries with clinical practice [33,34]. This trajectory mirrors, in many respects, the early phase of acute burn research in the 1990s and 2000s, when foundational insights into shock, infection, and hypermetabolism laid the groundwork for modern intensive care protocols [35]. Today, a similar process is unfolding in the realm of chronic burn outcomes, with translational studies now seeking to define biomarkers, pathophysiological models, and therapeutic strategies that target long-term dysfunction rather than acute survival alone [36,37].

### 3.2. Exemplary Development and Implementation: Burn-Associated Heart Failure

Among the emerging long-term complications, burn-associated heart failure (BAHF) represents one of the most striking and well-studied examples of this paradigm shift [38]. Initially identified through clinical observations of persistent cardiac dysfunction in long-term survivors and in large observational registries, the phenomenon has evolved from anecdotal reports into a systematically characterized entity [39,40]. Studies have shown that severe burns can induce lasting alterations in myocardial structure and function, including ventricular remodeling, reduced ejection fraction, and microvascular changes that persist long after wound healing [41]. In adolescent and young adult survivors of severe pediatric burns, these abnormalities have been accompanied by lower systolic and diastolic function than in healthy controls, reduced exercise tolerance, and evidence of myocardial fibrosis, with a substantial subset showing moderate-to-severe dysfunction years after injury. Clinically, this is important because many patients may remain only mildly symptomatic or subclinical at rest despite ongoing cardiac remodeling, raising the risk that progressive dysfunction will go unrecognized. Importantly, this complication is heterogeneous in both presentation and progression, ranging from subclinical myocardial dysfunction to overt, symptomatic heart failure that may develop months or years after the initial injury and persist for decades [41]. Together, these findings support the need for long-term cardiovascular surveillance in severe burn survivors, as such abnormalities may signal increased risk of early heart failure, later cardiovascular morbidity, and premature mortality.

The transition from acute cardiac stress to chronic myocardial disease reflects the chronification of an acute organ injury driven by sustained hypermetabolism, systemic inflammation, and neurohormonal dysregulation. Early cardiac involvement, once considered transient, has now been recognized as a potential precursor of long-term myocardial remodeling and functional decline. Evidence from longitudinal studies has demonstrated that subtle echocardiographic abnormalities and biomarker elevations in the early post-burn phase might be associated with later cardiac dysfunction, underscoring the need for systematic cardiac monitoring in long-term follow-up [21]. This resulted in the development of the CARABINIERI (CArdiac Response and Aftermath of Burn INjuries: Investigation of Etiology and Rehabilitative Interventions) Trial, which investigated long-term BAHF in adult burn survivors via echocardiography and cardiac magnet resonance imaging (Deutsches Register Klinischer Studien DRKS00014969).

At the mechanistic level, translational research has begun to bridge experimental and clinical insight. In vivo rat models of severe burn injury have been instrumental in delineating the molecular pathways of myocardial inflammation, fibrosis, and metabolic exhaustion, helping to uncover potential therapeutic targets [42]. One of the most promising translational applications has been the use of unspecific beta-blockers such as propranolol, which have been shown to mitigate hypermetabolism, reduce cardiac workload, and attenuate catabolic stress in both animal and clinical studies [43,44]. This approach exemplifies how pathophysiological understanding can translate into individualized therapy, turning mechanistic insight into practical, patient-centered intervention.

Despite this progress, the pathophysiology of burn-associated heart failure remains incompletely understood and is the subject of ongoing in vivo and clinical investigations exploring the interplay between systemic inflammation, oxidative stress, and neuroendocrine regulation [45]. Nonetheless, BAHF stands as a model paradigm for translational burn research, illustrating how acute organ dysfunction can evolve into chronic disease, how mechanistic understanding can inform therapy, and how this process can serve as a blueprint for investigating other organ systems affected by severe burns, such as skeletal muscle, liver, or the central nervous system [46,47].

**Figure 2:**
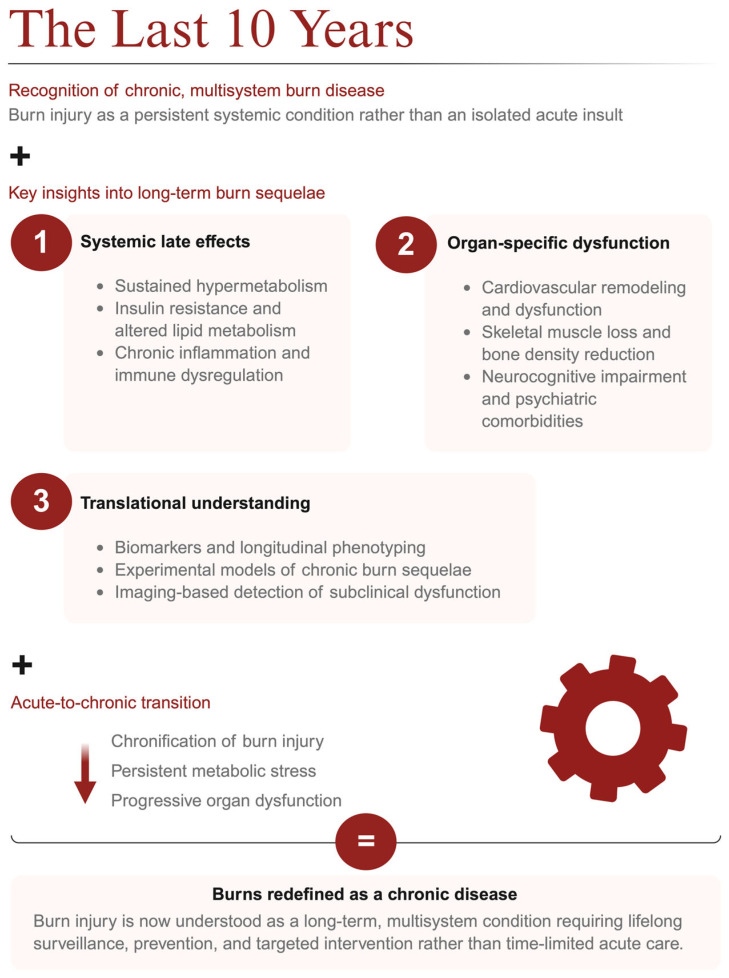
Over the last 10 years, burn injury has been redefined as a chronic, multisystem disease, with recognition of systemic late effects (persistent hypermetabolism, insulin resistance/altered lipid metabolism, chronic inflammation/immune dysregulation), organ-specific dysfunction (cardiovascular remodeling, muscle/bone loss, neurocognitive and psychiatric comorbidities), and growing translational tools (biomarkers/longitudinal phenotyping, experimental models, imaging of subclinical dysfunction) that illuminate the acute-to-chronic transition and the need for lifelong surveillance and targeted intervention.

In this sense, burn-associated heart failure not only defines a specific clinical entity but also symbolizes the broader transformation of burn medicine: from short-term survival toward the prevention, detection, and management of chronic, multisystem sequelae that define life after burn injury (Figure 2).

## 4. The Next 25 Years: Challenges and Perspectives

### 4.1. Identifying and Characterizing Unknown Long-Term Sequelae

As burn medicine advances further into the era of long-term survivorship, one of the foremost challenges for the coming decades will be to systematically identify and characterize the still-unknown long-term consequences of severe burn injury. Despite growing evidence of chronic multisystem involvement, many organ-specific and molecular effects remain poorly understood [21]. Establishing long-term cohort studies and national as well as international registries will be essential to capture the full spectrum of post-burn morbidity across diverse populations and time frames [48]. Future research will need to adopt a multiorgan and systems-based perspective, integrating cardiology, endocrinology, immunology, and neuroscience to unravel the interconnected physiological pathways that sustain post-burn alterations [49,50,51]. Such interdisciplinary collaborations will not only refine our understanding of chronic outcomes but also define the next generation of evidence-based interventions.

In this context, increasing attention must be directed toward interorgan cross-talk mechanisms that may contribute to the persistence and progression of long-term sequelae. Emerging evidence suggests that sustained communication between injured tissue, immune cells, liver metabolism, skeletal muscle, and the cardiovascular system may perpetuate chronic inflammation, metabolic dysregulation, and organ remodeling long after wound closure [30,52]. Disruption of these regulatory networks may therefore represent a key driver of post-burn chronification, underscoring the need for integrative research approaches that move beyond isolated organ analysis toward a systems-level understanding of long-term burn pathology.

### 4.2. Advancing Translational Research

A central scientific goal for the next 25 years should be to translate molecular and mechanistic insights into targeted therapeutic strategies. As research continues to reveal the cellular and biochemical underpinnings of chronic burn pathology, the field must move from descriptive observation to interventional precision. This will require the integration of biomarker discovery, advanced imaging modalities, and functional diagnostics to detect early organ dysfunction and guide personalized treatment [53,54].

For example, the identification of persistent mitochondrial dysfunction and oxidative stress pathways in long-term burn survivors has opened the door to potential mitochondria-targeted pharmacotherapies and antioxidant interventions, aiming to mitigate chronic metabolic and cardiovascular sequelae [55,56]. Similarly, functional cardiac MRI and advanced echocardiographic strain analysis are now being explored as imaging tools to detect subclinical myocardial impairment before symptomatic heart failure develops [57].

In parallel, translational advances in regenerative medicine are redefining the future of burn wound coverage and skin restoration. The development of bioengineered dermo-epidermal skin substitutes, such as denovoSkin^TM^, represents a significant milestone in the pursuit of physiological skin regeneration [58]. These autologous tissue-engineered constructs aim to overcome the limitations of conventional split-thickness skin grafting by restoring both epidermal and dermal components, thereby promoting improved vascularization, mechanical stability, and long-term functional outcomes. Ongoing clinical and experimental research focuses on optimizing cellular composition, matrix architecture, and host integration, with the objective of reducing hypertrophic scarring, improving elasticity, and achieving more durable and biologically representative skin repair [59]. In addition, vacuum-assisted closure (VAC) or negative-pressure wound therapy (NPWT) has become an important adjunct in burn care by reducing edema and exudate, improving perfusion, and promoting granulation tissue formation. When used over grafts or dermal substitutes, VAC therapy can help stabilize the wound bed and enhance graft take by providing uniform contact and decreasing shear forces [60,61,62].

Beyond local tissue regeneration, increasing attention is being directed toward the systemic metabolic regulation of burn recovery. Emerging evidence indicates that endocrine signaling pathways play a central role in coordinating post-burn inflammation, energy homeostasis, and tissue repair [63,64]. In this context, fibroblast growth factors (FGFs), particularly metabolically active subtypes such as FGF19 and FGF21, have gained interest as key regulators of hepatic glucose and lipid metabolism, mitochondrial function, and systemic energy balance. Dysregulation of FGF-mediated liver metabolism following severe burn injury may contribute to prolonged hypermetabolism, hepatic dysfunction, and impaired substrate availability for effective wound healing [65,66,67]. Elucidating these pathways may therefore identify future therapeutic targets aimed at metabolic modulation, linking systemic recovery with improved regenerative capacity.

Model organisms and long-term cell culture systems, such as murine models simulating chronic post-burn hypermetabolism and cardiac dysfunction or human 3D tissue-engineered skin and cardiac co-culture systems, will serve as indispensable platforms for preclinical investigation [68]. These models can replicate the persistent inflammatory and fibrotic environment of the post-burn state, thereby helping to bridge the gap between laboratory discovery and clinical application. The future of burn research will thus depend on fostering a translational continuum that links molecular characterization, mechanistic modeling, and therapeutic innovation within a unified research framework.

### 4.3. Establishing Lifelong, Multidisciplinary, Individualized Care

Beyond scientific discovery, the next generation of burn care must prioritize lifelong, individualized treatment pathways that address the medical, psychological, social, and functional dimensions of recovery. Holistic models of care, spanning acute management, rehabilitation, reconstructive interventions, and psychosocial reintegration, will form the cornerstone of this evolution [69]. Within these pathways, reconstructive surgery should be explicitly recognized as a longitudinal process that may require repeated, staged procedures and the full reconstructive ladder, from minimally invasive scar modulation and contracture release to flap-based reconstruction and microsurgical free tissue transfer, to restore motion, sensation, contour, and stable coverage over time. This reconstructive spectrum is integral to “life restoration,” particularly for function-limiting contractures, exposed critical structures, and complex regional defects where durable solutions extend beyond grafting alone [70,71].

An example of such an approach is the implementation of structured, activity-oriented rehabilitation programs, such as activity-oriented rehabilitation and workplace-related musculoskeletal rehabilitation, established within the BG Trauma Center network in Germany. These programs combine medical follow-up, targeted physical therapy, and occupation-specific training to restore functional capacity and facilitate the professional reintegration of burn survivors. Similarly, digital rehabilitation platforms and telemedicine tools, for instance, virtual scar assessment systems and remote physiotherapy applications, are extending the reach of specialized care into patients’ homes, ensuring continuous, adaptive support long after discharge [72,73,74].

Integrated rehabilitation and follow-up programs, supported by multidisciplinary teams, will ensure that burn survivors receive comprehensive care tailored to their evolving needs over time. In Germany, the BG Trauma Centers will continue to serve as a model of specialization and continuity, demonstrating how centralized expertise and structured care networks can optimize long-term outcomes. Expanding this model internationally, through cross-border research collaborations, standardized rehabilitation frameworks, and digital knowledge exchange, could establish a new global standard for comprehensive, lifelong burn care [75].

### 4.4. Ethical Challenges, Triage, and Resource Allocation in Modern Burn Care

The increasing survival of burn patients and the growing complexity of burn care have brought ethical decision-making to the forefront of modern burn medicine, particularly in scenarios involving mass casualty burn incidents. Severe burn injuries demand highly specialized resources, including intensive care capacity, operative infrastructure, skin substitutes, and experienced multidisciplinary teams. In large-scale events, these resources may become rapidly overwhelmed, necessitating difficult triage decisions that extend beyond individual patient-centered care toward population-based ethical frameworks [69].

Determining treatment priorities based on burn size, associated trauma, age, comorbidities, and expected long-term outcomes poses profound moral and clinical challenges, especially when survival probabilities and quality-of-life considerations must be weighed simultaneously. Events such as the Crans-Montana fire disaster exemplify how even healthcare systems in high-resource countries can reach critical capacity limits within hours, exposing vulnerabilities in regional preparedness and coordination. These situations raise fundamental questions regarding whether burn care resources should be decentralized to ensure rapid local access or strategically centralized within specialized high-volume centers to optimize outcomes.

Future burn care systems must therefore develop transparent, ethically grounded triage protocols and scalable disaster response strategies that balance equity, utility, and medical feasibility. Central to this evolution will be the establishment of transregional and cross-border burn networks, enabling coordinated patient distribution, shared surge capacity, and standardized decision-making across neighboring countries [76]. Such cooperative frameworks are essential to prevent ethical burden from falling on individual clinicians and to ensure that, in times of crisis, life-saving resources are allocated according to predefined, ethically sound, and internationally harmonized principles rather than ad hoc necessity.

### 4.5. Cross-Sector and Societal Implications

The long-term evolution of burn medicine will also depend on addressing the structural, digital, and educational dimensions of care. Sustainable healthcare frameworks and funding mechanisms will be essential to support lifelong treatment programs and to ensure equitable access to specialized services [77,78,79]. Digitalization and telemedicine will play an increasingly pivotal role in long-term follow-up and rehabilitation, enabling remote monitoring, virtual consultations, and interdisciplinary collaboration across regional and national boundaries. These technologies will be particularly valuable for reaching patients living in rural areas without access to specialized burn centers, as well as patients in developing countries, where the availability of advanced burn care remains limited [80,81]. By facilitating continuous expert input and patient education regardless of geography, telemedicine can help narrow the gap between high-resource and low-resource settings.

In addition to digital innovations, community-based and psychosocial programs such as burn camps have emerged as powerful tools for supporting social reintegration, especially among pediatric and adolescent burn survivors [82]. These initiatives provide safe spaces for peer connection, psychological resilience-building, and the re-establishment of self-esteem, key components of holistic, lifelong recovery that extend beyond medical care [83]. Here, family support is considered beneficial [84].

The education and training of future generations of burn care specialists must also evolve to meet the growing complexity of the field. Modern burn medicine now spans acute critical care, reconstructive surgery, rehabilitation science, and psychosocial health, requiring targeted, interdisciplinary training curricula and structured fellowship programs. Integrating simulation-based learning, tele-education, and collaborative international exchanges could strengthen both clinical expertise and research capacity. Such programs should also foster leadership in digital health, translational research, and long-term care coordination, ensuring that future burn specialists are equipped not only to save lives but to guide survivors through the entire continuum of recovery [85,86,87].

Beyond professional education, increasing public awareness of burn risks and prevention strategies represents a critical societal responsibility. Many severe burn injuries remain preventable and are frequently associated with household accidents, occupational hazards, and insufficient safety training. Strengthening public education initiatives, improving workplace safety standards, and promoting early risk awareness may substantially reduce the incidence of life-altering burn injuries. In this context, prevention must be recognized as an integral component of comprehensive burn care, complementing medical innovation by addressing the societal determinants that precede injury itself.

**Figure 3:**
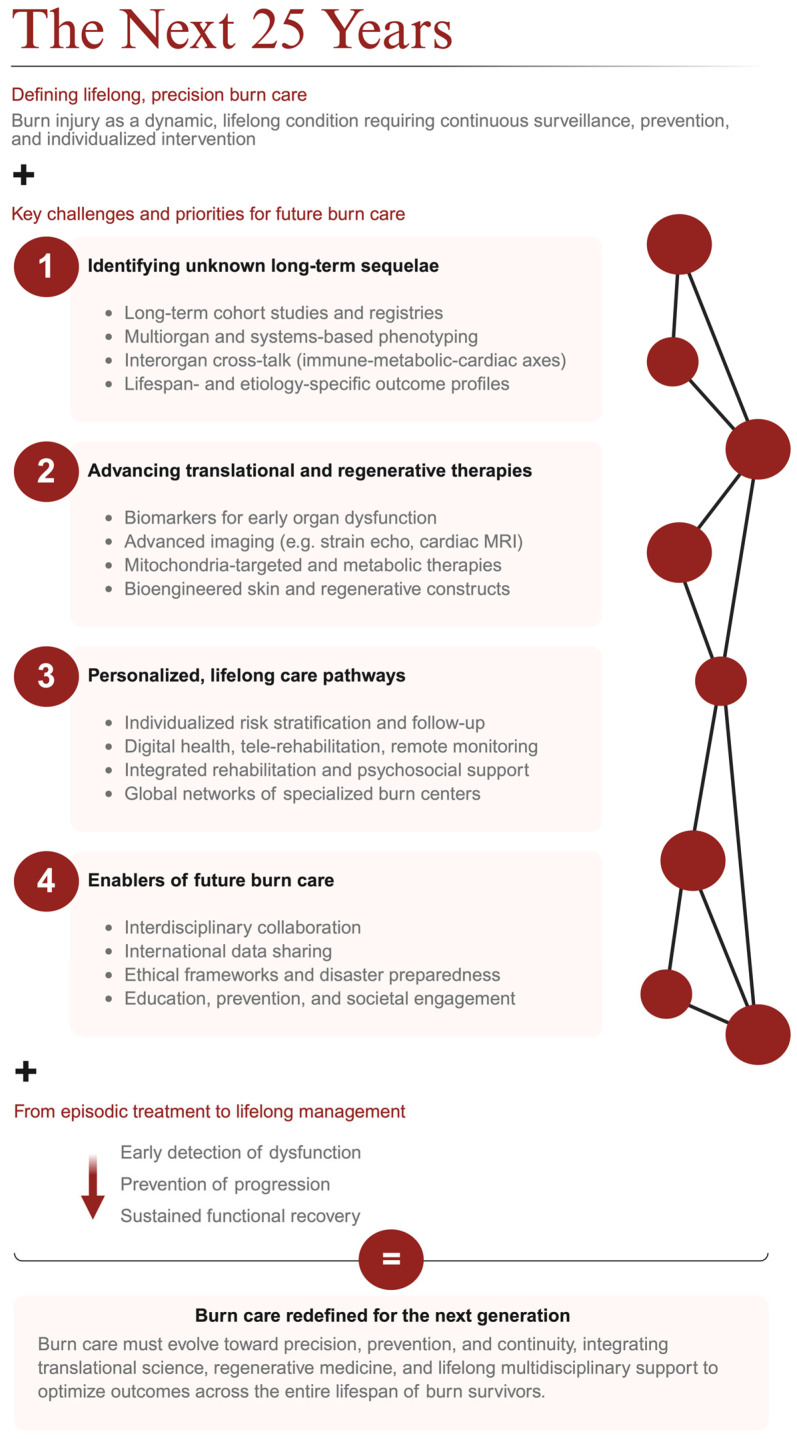
Framework for the next 25 years of burn care, emphasizing lifelong, precision management with continuous surveillance, prevention, and individualized intervention. Future priorities include (1) identifying unknown long-term sequelae through long-term cohorts/registries and systems-based phenotyping, (2) advancing translational and regenerative therapies (early biomarkers, advanced imaging, metabolic/mitochondrial targets, bioengineered skin constructs), (3) building personalized lifelong care pathways (risk stratification, digital/remote monitoring, integrated rehabilitation and psychosocial support, specialized burn-center networks), and (4) enabling progress via interdisciplinary collaboration, international data sharing, ethical frameworks/disaster preparedness, and education/prevention—shifting care from episodic treatment to early detection, prevention of progression, and sustained functional recovery.

Ultimately, the coming 25 years will challenge the field not merely to extend survival, but to redefine what it means to live well after a severe burn injury through equity of access, continuity of care, and a deeper understanding of the lifelong needs of burn survivors (Figure 3).

## 5. Conclusions and Outlook

Burn medicine stands at the beginning of a new era, an era of long-term and personalized burn care. As survival after severe burns has become an achievable standard, the discipline now faces the challenge of securing lifelong health, functionality, and quality of life for an expanding population of survivors. The future of burn care will therefore depend on the ability to integrate personalized medicine, interdisciplinary collaboration, and translational research into a cohesive continuum that extends from the acute phase to lifelong follow-up. At the same time, the present manuscript is limited by its narrative review methodology, which does not allow for a formal quantitative synthesis of the available evidence. Accordingly, the conclusions should be interpreted within the methodological boundaries of this manuscript type.

However, at the same time, different burn injury mechanisms require distinct treatment strategies and may lead to unique long-term sequelae that are only beginning to be understood. For instance, flame, scald, high-voltage electrical burns, and thermo-mechanical or explosion-related trauma often differ significantly in their systemic impact, tissue damage patterns, and chronic outcomes [88,89]. The latter, particularly, have gained relevance in recent years due to complex polytrauma patterns observed in military and civilian conflict zones, such as in Ukraine, with an increased number of acute and long-term burn patients presenting to specialized burn centers across Europe [90,91,92]. These evolving injury profiles underscore the need for differentiated research approaches, tailored rehabilitation pathways, and the systematic documentation of long-term consequences specific to each burn etiology.

Specialized centers, such as the BG Trauma Centers in Germany, will continue to play a pivotal role as catalysts of translational innovation and continuous care. Their integrated model, combining acute treatment, reconstructive surgery, rehabilitation, psychological support, and long-term monitoring, represents one of the blueprints for modern burn care systems worldwide. Expanding such structures into international networks will be essential for standardizing care, sharing data, and fostering global collaboration in both clinical and research domains [69]. However, the realization of this vision will depend heavily on strong interdisciplinary collaboration, the commitment of healthcare systems and governmental institutions, and sustained funding for clinical care, research, and education. Only through this joint effort, uniting clinicians, scientists, policymakers, and stakeholders, can the advances achieved in specialized centers truly translate into comprehensive, accessible, and equitable burn care on a global scale. Here, sustained investment in research and funding frameworks that enable long-term studies, biobanking, and multicenter clinical trials is necessary. Only through such coordinated efforts can the field translate emerging discoveries, from molecular mechanisms to therapeutic interventions, into tangible benefits for patients.

Ultimately, the vision for the next 25 years is clear: to move from survival toward sustained health, where burn care encompasses not only the rescue of life but also the restoration of its full physical, psychological, and social potential. Achieving this vision will require an unwavering commitment to scientific progress, interdisciplinary teamwork, equitable access to lifelong care, and a deeper understanding of the diversity and complexity of burn injuries that define the patients of tomorrow.

## Data Availability

Further relevant data is available from the corresponding author upon reasonable request.
